# Antibiotic genes spread far and wide

**DOI:** 10.7554/eLife.05244

**Published:** 2014-11-25

**Authors:** Ryan J Catchpole, Anthony M Poole

**Affiliations:** 1 **Ryan J Catchpole** is in the Biomolecular Interaction Centre, School of Biological Sciences, University of Canterbury, Christchurch, New Zealand; 2 **Anthony M Poole** is in the Biomolecular Interaction Centre, School of Biological Sciences, University of Canterbury, Christchurch, New Zealandanthony.poole@canterbury.ac.nz

**Keywords:** horizontal gene transfer, antibiotic, lysin, archaea, other

## Abstract

The genes responsible for antibiotics can spread between the three domains of life—Archaea, Bacteria and Eukaryotes.

**Related research article** Metcalf JA, Funkhouser-Jones LJ, Brileya K, Reysenbach AL, Bordenstein SR. 2014. Antibacterial gene transfer across the tree of life. *eLife* **3**:e04266. doi: 10.7554/eLife.04266.

**Image** A microbe from the Archaea domain produces an antibiotic that can kill bacteria

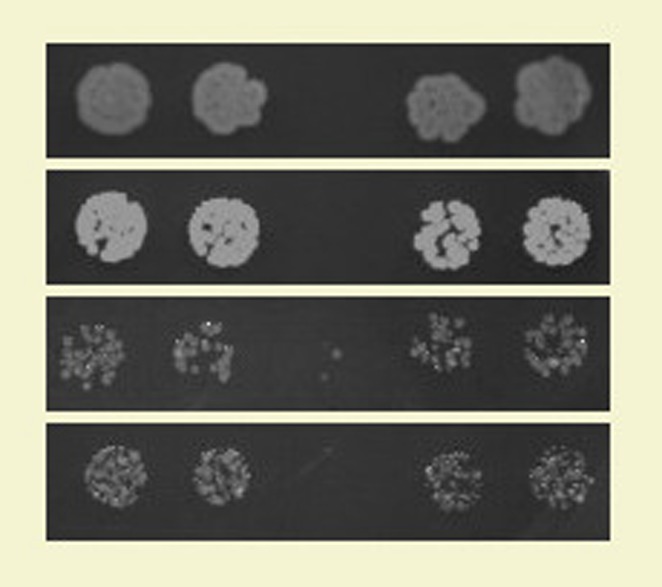

The development of antibiotics in the 1940s meant that, for the first time in history, bacterial infections were readily treatable. However, their widespread use since then has resulted in the spread of bacteria that are resistant to many conventional antibiotics (Laxminarayan, 2014). In the United States alone, antibiotic-resistant bacteria infect at least 2 million people each year, and result in over 23,000 deaths. However, the design of new antibiotic drugs, while promising, is slow and expensive and it can be difficult to identify new therapeutic compounds, not least because agents as diverse as enzymes and ions can have antibacterial properties.

Now, in *eLife*, Seth Bordenstein of Vanderbilt University, Anna–Louise Reysenback of Portland State University and co-workers—including Jason Metcalf as first author—reveal that the genes for proteins with antibacterial properties are capable of spreading across stunning evolutionary distances (Metcalf et al., 2014). Their results suggest that our search for new antibiotics needs to be broadened if we are to take full advantage of the variety of antibacterial compounds that exist in nature.

Genes are able to move between organisms in a process known as horizontal gene transfer. This happens most frequently between individuals of the same, or closely-related species (Andam and Gogarten 2011) and is thought to be responsible for the spread of antibiotic resistance genes between bacteria. However, genes can also occasionally move between distantly-related individuals, including from one domain of life, such as Bacteria, to either Archaea or Eukaryotes (Lundin et al., 2010). Whether a gene is successfully transferred depends on a number of constraints. For instance, if the organisms inhabit different environments, there are fewer opportunities to transfer genes. Once transferred, a gene may not be compatible with the recipient, or may not provide it with an advantage. Despite these constraints, some genes have spread, via horizontal transfer, to all three domains of life, and such transfer events may have been extensive during evolution (Nelson-Sathi et al., 2014).

Metcalf et al. used bioinformatic techniques to reveal that genes belonging to the lysozyme family have been involved in at least three independent gene transfers between domains. Lysozyme is an enzyme that acts as an antibacterial because it degrades the cell walls of bacteria, making cells susceptible to rupture (Callewaert et al., 2012). As a result of these gene transfers, lysozyme has been integrated into the antibacterial armouries of organisms from all three domains. This is encouraging because it suggests that antibiotic genes can spread just as effectively as the genes for antibiotic resistance, so it is likely that there are many potential new therapeutic agents still to be discovered.

Bordenstein and colleagues identified a lysozyme gene in the single-celled microbe *Aciduliprofundum boonei*, which is particularly interesting as it is the first example of an antibacterial gene in the domain Archaea. They showed that purified extracts of the archaeal lysozyme have antibacterial activity in vivo. Lysozyme production is increased when A. *boonei* is co-cultured with the bacterium *Mesoaciditoga lauensis*, suggesting that *A. boonei* produces lysozyme in response to bacterial competition. This is significant because, when grown in pure culture, *A. boonei* grows slower than a bacterial competitor from the same environment. However, when grown together on the same medium, the tables are turned, and *A. boonei* grows faster than *M. lauensis*. This suggests that lysozyme may be acting to inhibit the growth of the bacterium, giving *A. boonei* a competitive advantage.

The competitive ability that lysozyme imparts on *A. boonei* suggests that archaea and bacteria may not live together as harmoniously as once thought. Rather, some archaea may respond to the presence of bacterial competitors in a hostile manner. The work of Metcalf et al. thus significantly advances our understanding of interactions in microbial communities and suggests new avenues to search for potential antibiotics. Since the bacteria that cause diseases in humans and animals exist in a wide range of environments, the other microbes that live alongside them may be an untapped source of antibiotics (Martinez, 2008).

Given the importance of antibiotics to human health, this study shows that, in our search for effective antibiotics, we would do well to embrace the diversity of life and spread our search wide. Seen in this light, genome sequencing projects driven by biodiversity, such as the Genomic Encyclopedia of Bacteria and Archaea (Wu et al., 2009; Rinke et al., 2013), provide greater value than merely sating our curiosity: they may also help to uncover novel antibiotics from unlikely sources.
